# Baicalin induces apoptosis and autophagy in human osteosarcoma cells by increasing ROS to inhibit PI3K/Akt/mTOR, ERK1/2 and β-catenin signaling pathways

**DOI:** 10.1016/j.jbo.2022.100415

**Published:** 2022-02-01

**Authors:** He Pang, Tingrui Wu, Zhonghua Peng, Qichao Tan, Xin Peng, Zeyu Zhan, Lijun Song, Bo Wei

**Affiliations:** a Orthopedics Center, Affiliated Hospital of Guangdong Medical University, Zhanjiang 524001, China; b Reproductive Medicine Center, Affiliated Hospital of Guangdong Medical University, Zhanjiang 524001, China

**Keywords:** Baicalin, Osteosarcoma, Autophagy, Apoptosis, ROS, PI3Kγ

## Abstract

Baicalin, a flavonoid derivative, exerts antitumor activity in a variety of neoplasms. However, whether baicalin exerts antitumor effects on osteosarcoma cells remains to be elucidated. In this study, treatment with baicalin reduced the proliferation and invasive potential of osteosarcoma cells and reduced the mitochondrial membrane potential, which eventually caused mitochondrial apoptosis. In addition, baicalin increased intercellular Ca^2+^ and ROS concentrations. Baicalin-induced apoptosis was confirmed by enhanced Bax, cleaved caspase-3, and cleaved PARP levels and decreased Bcl-2 levels. The increase in LC3-II and p62 suggested that baicalin induced autophagosome formation but ultimately inhibited downstream autophagy. Moreover, apoptosis induced by baicalin was attenuated by the addition of 3-MA. Furthermore, we found that baicalin inhibited the PI3K/Akt/mTOR, ERK1/2 and β-catenin signaling pathways. Chelation of free Ca^2+^ by BAPTA-AM also inhibited both apoptosis induction and ROS concentration changes. Finally, NAC pretreatment reversed baicalin treatment outcomes, including the increase in Ca^2+^ concentration, induction of apoptosis and autophagy, and inhibition of the pathways. Molecular docking results indicated that baicalin might interact with the structural domain of PI3Kγ. Thus, baicalin may be considered a potential candidate for osteosarcoma treatment.

## Highlights

• Baicalin causes apoptosis and autophagy through accumulating ROS to suppress PI3K/Akt/mTOR, ERK1/2 and β-catenin pathways in OS cells.
• Baicalin-induced autophagosome further triggers apoptosis.
• Baicalin-induced ROS and Ca^2+^ interactions induce apoptosis.
• Baicalin molecule targets PI3Kγ, inhibiting downstream effectors AKT and mTOR.

## 1 Introduction

Osteosarcoma (OS) is a common malignant bone cancer with the highest incidence in adolescents and occurs in the epiphysis of long bones[1], [2]. The median age of osteosarcoma patients is 16 years, which appears to be associated with growth spurts [3], [4]. At present, despite advancements in chemotherapy and surgery, the 5-year survival rates for primary and metastatic osteosarcoma remain<65% and 25%, respectively, with few treatment options [5], [6]. Thus, it is essential to explore practical and safe therapeutic agents for osteosarcoma. Baicalin, a flavonoid derivative compound, is obtained from the root of Scutellaria. In recent reports, baicalin was found to possess antioxidant and anti-inflammatory properties and is virtually nontoxic to healthy tissues [7], [8]. Data indicate that baicalin inhibits tumor growth and metastasis in various cancer cells [9], [10], [11]. These effects are transduced by reactive oxygen species-mediated mitochondrial pathways and via inhibition of the AKT pathway [12], [13], [14], [15]. Although the mechanism underlying baicalin’s inhibition of osteosarcoma cells can partially be attributed to autophagy, apoptosis and the suppression of multiple signaling pathways, their interplay remains unclear. Therefore, understanding baicalin-mediated changes and their underlying mechanisms may lead to identification of an effective candidate for osteosarcoma treatment.

Disturbed regulation of the cell cycle can promote the occurrence and development of tumors [16], [17]. Many cytotoxic agents have been found to cause cell cycle S-phase arrest, promoting cell death [18], [19]. The characteristic manifestations of apoptosis are nuclear fragmentation, cell shrinkage and apoptotic vesicle development. [20], [21]. In particular, a recent report showed that numerous chemotherapeutic agents regulate the family members of Bcl-2 to induce mitochondrial apoptosis [22]. The Bcl-2 protein family is considered to be the crucial factor controlling mitochondrial membrane permeabilization. Apoptosis has played a significant role in osteosarcoma treatment during the past three decades, and elemental signaling pathways have been extensively investigated. The cell death mechanism type II, known as autophagy, which is a “self-feeding” process, includes the formation of autophagic vesicles and the decomposition of cytoplasmic components [23]. Autophagy plays a dual role in tumorigenesis and progression because it promotes cell survival to prevent apoptosis in some cellular environments, while it induces cell death in others [24], [25]. The association between autophagy and apoptosis is complex due to differences in cell types and environmental stimuli. Autophagy is activated and regulated by reactive oxygen species and Ca^2+^ concentration [26], [27]. Recent research has shown that a significant number of apoptosis-inducing cancer chemotherapy drugs also activate autophagy [28], [29]. Whether baicalin induces apoptosis or autophagy remains to be determined.

Endogenous and exogenous factors activate reactive oxygen species (ROS), which are involved in regulating biological activity [30]. In the presence of external stimuli beyond a certain degree, the production of excess ROS damages cellular integrity due to a disrupted balance between the generation and metabolism of ROS [31]. The damage of ROS to cells is primarily reflected in the damage to biomacromolecules, such as oxidative damage to DNA, proteins, and biofilms [32]. An increasing number of reports indicate that reactive oxygen species have a critical function in the induction of apoptosis and autophagy [33], [34]. Moreover, reactive oxygen species affect multiple signaling pathways [35]. Recent studies have shown that ROS regulate the PI3K/Akt/mTOR, ERK1/2 and β-catenin signaling pathways. These pathways impact the cell cycle, apoptosis, invasion and autophagy induction [36], [37], [38]. Intracellular calcium concentration is intimately linked to reactive oxygen species generation [39]. Extensive investigations have revealed that elevated intercellular Ca^2+^ levels induce endoplasmic reticulum stress and constitute a significant proapoptotic signal [40]. In addition, Ca^2+^ is also a stimulator of apoptosis and autophagy [41], [42]. In this study, we explored the anti-osteosarcoma mechanism of baicalin in vitro. In addition, we investigated baicalin-induced cell cycle arrest, cell death and potential signaling pathways.

## 2 Materials and methods

### 2.1 Cell culture

The HOS, MG63 U2OS and 143B human osteosarcoma cell lines (Chinese Academy of Sciences Cell Bank) were cultured in MEM containing 10% fetal bovine serum (Gibco, Carlsbad, USA) and 0.5% penicillin and streptomycin (Gibco, Sydney, Australia) and maintained in a cell incubator with 95% humidity at 37 °C and 5% carbon dioxide.

### 2.2 Antibodies and reagents

Baicalin (purity > 98%) (Shanghai Yuanye Biotechnology, Shanghai, China) was dissolved in DMSO. DMSO (<0.5%) was added to the control group as a vehicle control. N-Acetyl-L-cysteine (NAC) was obtained from Beyotime Institute of Biotechnology. 3-Methyladenine (3-MA) was obtained from Solarbio Biotechnology (Beijing, China). Antibodies against Bcl-2(D55G8), Bax (D2E11), cleaved PARP (Asp214), cleaved caspase-3 (Asp175), p-ERK1/2 (D13.14.4E), p-AKT (D25E6), β-catenin (D10A8), AKT (C67E7), p-mTOR (D9C2), mTOR (7C10), GAPDH (D16H11), c-Myc (E5Q6W), Cdk2 (E8J9T), cyclinE1 (D7T3U), cyclinA2 (E6D1J) and the secondary antibodies (goat anti-rabbit IgG and anti-mouse IgG) were purchased from Cell Signaling Technology (Beverly, MA, USA). Caspase-3 (ab13847), LC3 (ab48394) and p62 (ab56416) antibodies were obtained from Abcam Biotechnology. Antibodies against ERK1/2 (C-9) were obtained from Santa Cruz Biotechnology.

### 2.3 Cell viability assay

Cells were seeded into 96-well plates at approximately 100 μL and 2 × 10^3^ cells per well, cultured for 24 h, and then treated with different concentrations (0, 20, 40, 80 μM) of baicalin for 24–72 h. Cell viability was measured using Cell Counting Kit-8 (CCK-8) reagent (Beyotime, Shanghai, China). After incubation for 2 h, the absorbance of the cells was measured at 450 nm.

### 2.4 Colony formation assay

HOS, MG63, U2OS, and 143B osteosarcoma cells (approximately 800 cells/well) were cultured in 6-well plates for two weeks with different concentrations of baicalin. At the end of cell culture, cells were fixed in methanol for 15 min and then stained with crystal violet staining solution at room temperature for 10 min. Colonies containing > 50 cells were counted under a microscope.

### 2.5 Invasion assay

A chamber was coated with Matrigel matrix (Corning) and placed in an incubator for 30 min. Then, 100 µL of MEM containing a total of 2 × 10^4^ cells was added. After incubation for 24 h, we removed cells that did not penetrate the membrane inside the chamber. Cells inside the membrane were immobilized with methanol for 15 min and then stained with crystal violet staining solution at room temperature for 10 min. The average cell numbers of six microscopic fields of view (100 × ) were counted using ImageJ software.

### 2.6 Cell cycle analysis

HOS and 143B cells were treated with baicalin for 48 h. Next, cells were collected and fixed in frozen ethanol for 12 h. Next, cells were stained with propidium iodide (PI) (Beyotime, Shanghai, China) for 30 min at room temperature and then analyzed by flow cytometry.

### 2.7 Apoptosis analysis by flow cytometry

Apoptosis was evaluated using the Annexin V-FITC/PI Apoptosis Detection Kit (Beyotime, Shanghai, China). HOS, MG63, U2OS, and 143B cells were trypsinized after different treatments and collected. Then binding buffer was added to the cells, and they were incubated in the dark for 30 min before evaluation by flow cytometry.

### 2.8 Mitochondrial membrane potential (MMP) assay

Osteosarcoma cells were subjected to various treatments for 48 h. Subsequently, we collected and washed the cells. Next, the JC-1 working solution of the MMP Assay Kit (Beyotime, Shanghai, China) was used to treat the cells for 30 min, which were finally examined by flow cytometry. JC-1 is widely used as an ideal fluorescent probe for detecting mitochondrial membrane potential. The mitochondrial membrane potential of normal cells is high, so JC-1 accumulates in the matrix of mitochondria and form polymers (J-aggregates), resulting in the production of red fluorescence. In contrast, when the mitochondrial membrane potential of cells is low, JC-1 cannot accumulate in the matrix of mitochondria, resulting in increased green fluorescence, allowing convenient detection of changes in mitochondrial membrane potential through the transition of fluorescence color.

### 2.9 ROS assay

The ROS Assay kit (Beyotime, Shanghai, China) was used to assess intracellular reactive oxygen species production. Osteosarcoma cells were subjected to different treatments for 48 h. Subsequently, the cells were collected and stained with 10 μM DCFH-DA for 30 min while protected from light and eventually measured by flow cytometry.

### 2.10 Ca^2+^ assay

To analyze intracellular Ca^2+^ concentrations, approximately 2 μM of the Ca^2+^ indicator Fluo-4 AM (Beyotime, Shanghai, China) was loaded into HOS and 143B cells, and the cells were then incubated with a fluorophore for 30 min. Finally, the Ca^2+^ content was estimated using flow cytometry.

### 2.11 Western blot analysis

Total protein was extracted from cells in each group. Protein concentration was measured and subjected to gel electrophoresis. After membrane transfer and blocking, we added a primary antibody overnight at 4 °C. Then, the membrane was washed with TBST and incubated with secondary antibody. Finally, immunoreactive bands were measured using a chemiluminescence and fluorescence imaging system. The intensity of the target protein bands was normalized to the intensity of GAPDH bands, and ImageJ software was used to perform band grayscale analysis.

### 2.12 Molecular docking

To investigate the interaction between baicalin and the PI3Kγ active site, Discovery Studio software (DS 2019, Accelrys, CA, USA) and AutoDock Vina1.1.2 software were used to conduct automated molecular docking. The three-dimensional crystal structure of PI3K gamma (PI3Kγ) was complexed with the inhibitor from PDB (PDB ID: 4XZ4). PyMOL 2.3.0 software was used to remove protein crystalline water, primitive ligands, etc. Subsequently, the PI3Kγ structure was loaded into AutoDocktools (v1.5.6) software for hydrogenation, charge assignment and designation of atoms. Molecular docking was conducted using AutoDock Vina1.1.2 software, and finally, the molecular docking results were imported into Discovery Studio2019 for visualization.

### 2.13 Statistical analysis

All data are expressed as the mean ± SD of at least three independent experiments. GraphPad Prism 8.0 software (California, USA) was used for data analysis. Student's *t* test or one-way ANOVA was used to analyze differences between groups. P*＜ 0.05 were considered statistically significant.

## 3 Result

### 3.1 Baicalin inhibits proliferation and induces S-phase blockade in osteosarcoma cells

**Fig. 1:**
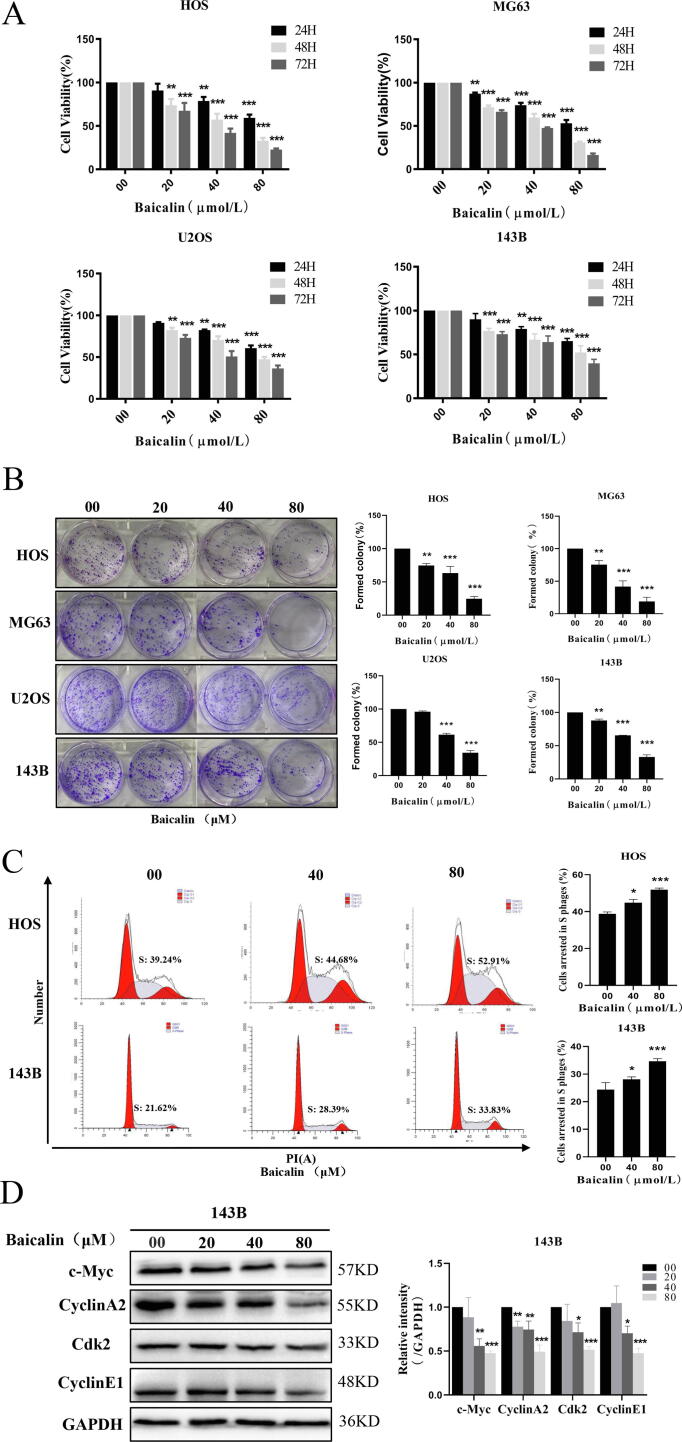
**Baicalin represses proliferation of OS cells. (A)** HOS, MG63, U2OS and 143B cells were incubated with different concentrations of baicalin for 24–72 h. Cell viability was quantified using a CCK-8 assay. **(B)** Colony formation after baicalin treatment was evaluated as determined by the colony formation assay. **(C)** Baicalin-induced S-phase cell cycle arrest was detected by flow cytometry. **(D)** Expression of c-Myc, cyclin A2, Cdk2 and cyclinE1was analyzed by western blotting. *p < 0.05, **p < 0.01, ***p < 0.001 vs. control group.

To investigate the antiproliferative activity of baicalin on osteosarcoma, the human osteosarcoma cell lines HOS, MG-63, U2OS, and 143B were treated with different concentrations of baicalin for 24, 48 or 72 h. Then, cell viability assays revealed that baicalin suppressed osteosarcoma cell proliferation in a concentration- and time-dependent manner (Fig. 1A). Moreover, colony formation assays demonstrated that baicalin treatment decreased colony formation (Fig. 1B). The above data indicated that baicalin reduced the proliferation of osteosarcoma cells. To investigate the relationship between the anti-osteosarcoma effects of baicalin and cell cycle blockade, we analyzed the influence of baicalin on cell cycle progression. After treatment with different concentrations of baicalin (0, 40, or 80 μM) for 48 h, the percentages of S phase cells were 38.8 ± 1.0%, 44.9 ± 1.8%, and 51.9 ± 0.9% in HOS cells and 24.4 ± 2.6%, 28.1 ± 0.9%, and 34.7 ± 0.9% in 143B cells (Fig. 1C). These data indicate that the percentage of HOS and 143B cells in S phase was increased in response to baicalin treatment. Moreover, western blotting results revealed that baicalin treatment significantly decreased c-Myc, cyclin A2, Cdk2 and cyclin E1 expression levels. In summary, these results showed that baicalin causes cell cycle blockade at the S-phase and inhibits proliferation by interfering with cell cycle- and proliferation-related proteins.

### 3.2 Baicalin inhibits the invasive ability of osteosarcoma cells

**Fig. 2:**
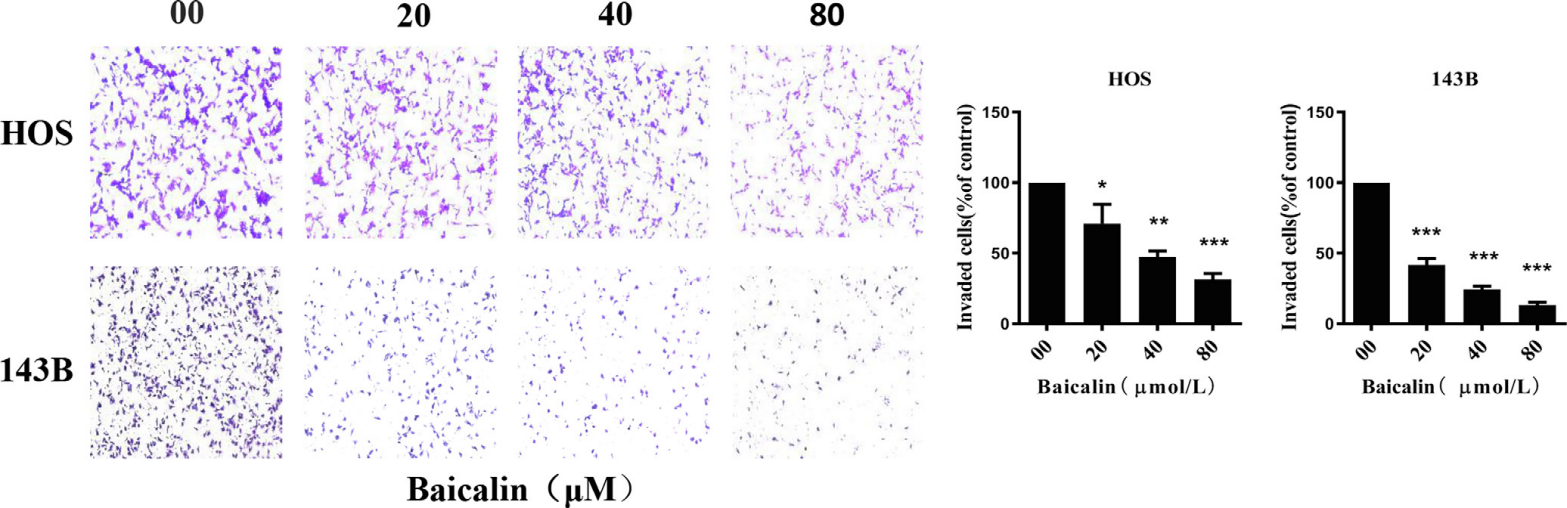
**Baicalin suppresses invasion in OS cells.** Invasion assays assessed the invasion ability of osteosarcoma cells. The number of invasive cells in five independent microscopic fields was quantified using ImageJ. *p < 0.05, **p < 0.01, ***p < 0.001 vs. control group.

Invasion assays were performed to determine the invasion of HOS and 143B cells. Osteosarcoma cells were treated with baicalin for 24 h. Then, cells penetrating the polycarbonate membrane were observed by microscopy at 100 × magnification. The results of the cell invasion assay revealed that the number of invasive cells (% compared to the control group) of HOS and 143B cells was 31.3 ± 7.4% and 13.3 ± 3.2% after treatment with 80 µmol/L baicalin for 24 h (Fig. 2). Overall, our findings reveal that baicalin inhibits the invasive ability of osteosarcoma cells.

### 3.3 Baicalin induces mitochondria-mediated apoptosis in osteosarcoma cells

**Fig. 3:**
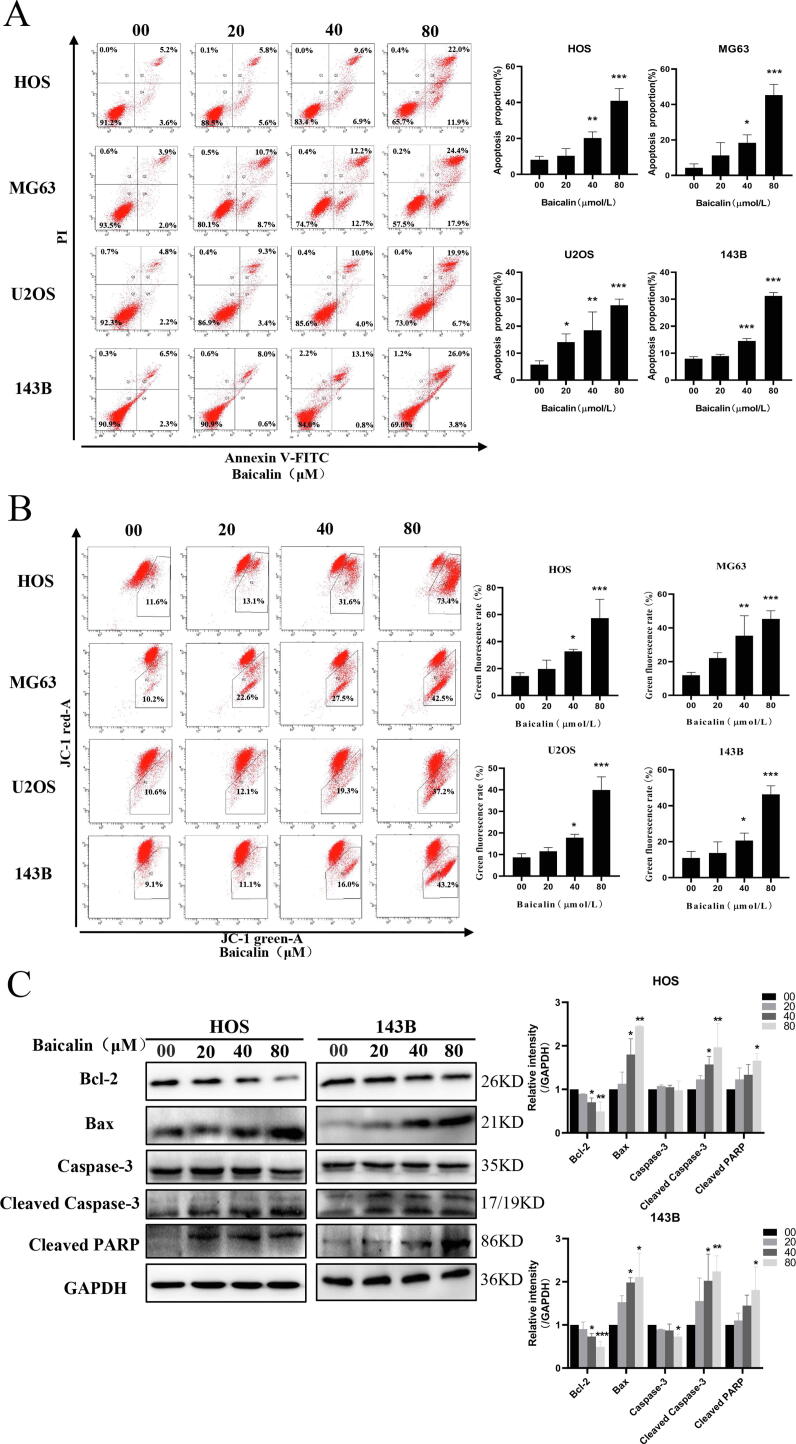
**Baicalin induces apoptosis in osteosarcoma cells. (A)** The percentage of OS cells undergoing apoptosis was evaluated by flow cytometry analysis. **(B)** MMP was subjected to flow cytometry analysis using the fluorescent mitochondrial probe JC-1. **(C)** Bcl-2, Bax, caspase-3, cleaved caspase-3 and cleaved PARP expression was examined by western blotting. *p < 0.05, **p < 0.01, ***p < 0.001 vs. control group.

Commonly, cell cycle inhibition is closely associated with apoptosis. Hence, baicalin-induced apoptosis was further investigated using a flow cytometry assay. To quantify apoptosis, we used an Annexin V-PI double staining method. After treatment with different concentrations of baicalin for 48 h, the percentages of apoptotic HOS cells were 8.1 ± 2.0%, 10.4 ± 4.0%, 20.1 ± 3.5% and 40.9 ± 6.8%. The percentage of apoptosis was 4.3 ± 2.2%, 11.2 ± 7.2%, 18.3 ± 4.5% and 45.3 ± 6.1% in MG63 cells. The percentages of apoptosis were 5.8 ± 1.4%, 14.1 ± 3.1%, 18.5 ± 6.8%, and 27.8 ± 2.3% in U2OS cells. The percentages of apoptotic 143B cells were 8.0 ± 0.7, 8.9 ± 0.7, 14.5 ± 0.9 and 31.2 ± 1.3%. These results demonstrated that osteosarcoma cells displayed an increase in the apoptotic cell ratio in response to baicalin treatment for 48 h (Fig. 3A). A decrease in mitochondrial membrane potential is a hallmark event in the early stages of apoptosis. To explore the influence of baicalin on mitochondrial membrane potential, we measured MMP using JC-1 working solution and flow cytometry analysis. The transition of JC-1 from red fluorescence to green fluorescence indicates a decrease in mitochondrial membrane potential. At the same time, this transition can be used as a detection indicator of the early stage of apoptosis. After treatment with different concentrations of baicalin for 48 h in HOS, MG63, U2OS and 143B cells, the rate of green fluorescence increased to different extents, and the rate of green fluorescence in the 80 µmol/L baicalin treatment group was 57.4 ± 14.0%, 45.4 ± 4.8%, 40.0 ± 6.1% and 46.4 ± 4.8%, respectively. These results indicate that the green fluorescence intensity ratio increased after baicalin treatment for 48 h as determined by flow cytometry (Fig. 3B), suggesting that baicalin induces apoptosis in osteosarcoma cells by decreasing MMP and impairing the integrity of the mitochondrial membrane. Subsequently, we further assessed apoptosis-associated proteins. These results demonstrated that Bcl-2 was sharply reduced in cells after baicalin treatment, while Bax, cleaved caspase-3 and cleaved PARP gradually increased (Fig. 3C). These findings indicate that baicalin induces apoptosis through a mitochondria-mediated apoptotic pathway.

### 3.4 Baicalin triggers autophagy in osteosarcoma cells

**Fig. 4:**
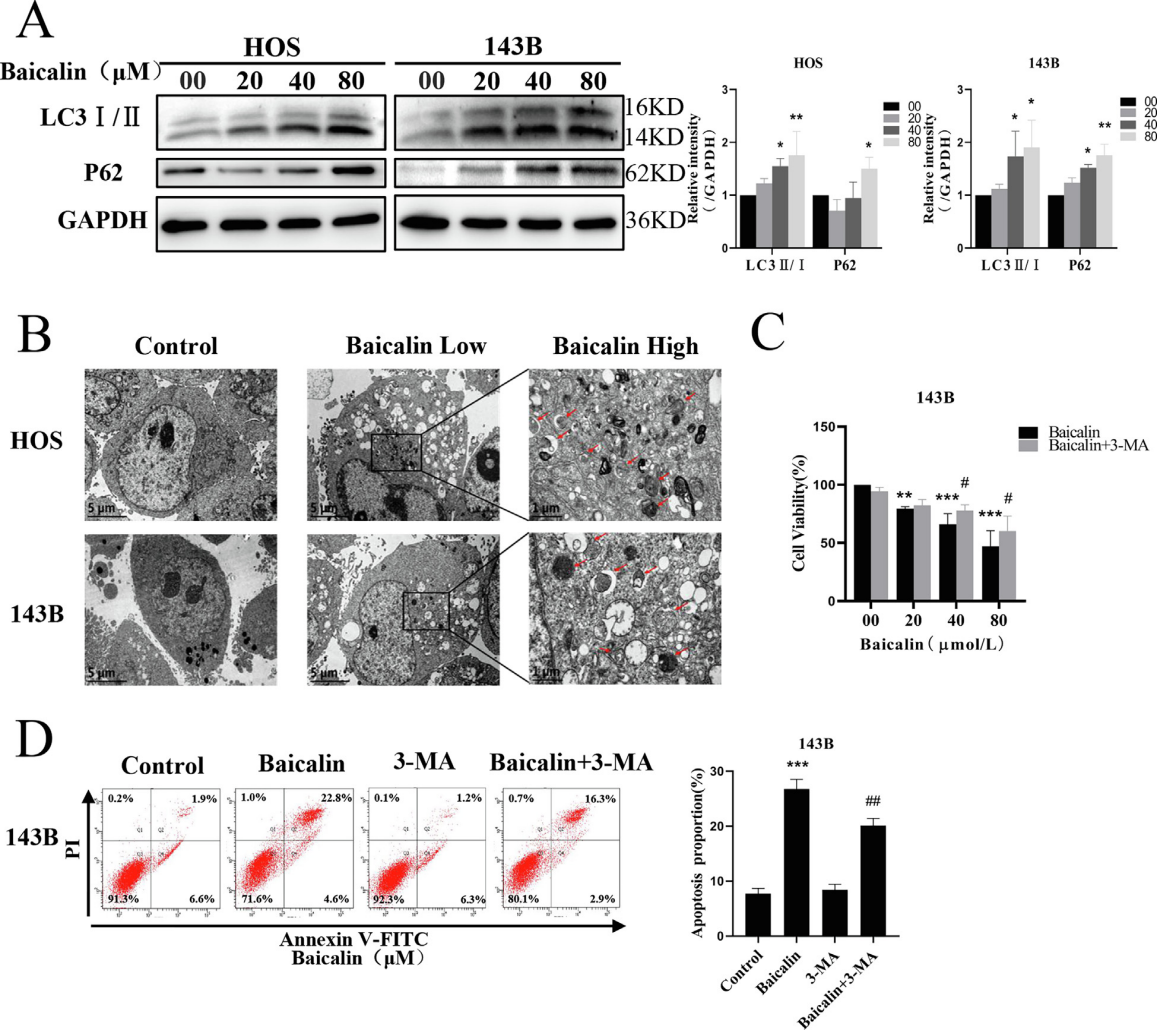
**Baicalin triggers autophagy in osteosarcoma cells. (A)** Western blot analysis showing LC3-I/II and p62 expression in 143B and HOS cells after baicalin treatment. **(B)** Detection of autophagosomes in osteosarcoma cells by transmission electron microscopy. Low: 8000X; High: 30,000X. 143B cells were pretreated with 3-MA (3 mM) for 2 h followed by treatment with baicalin for 48 h. **(C)** Cell proliferation was analyzed by cell viability assay. **(D)** The apoptosis rate was detected by flow cytometry analysis. *p < 0.05, **p < 0.01, ***p < 0.001 vs. control group. **^#^***p* < 0.05 vs. baicalin-treated group.

To investigate whether autophagy participates in baicalin-induced apoptosis, we analyzed the effect of baicalin on autophagy in osteosarcoma cells. LC3-II and P62 are markers of autophagosomes and autophagic activity, respectively. The results indicated that levels of LC3-II and p62 were increased by baicalin treatment (Fig. 4A). Autophagosomes observed by electron microscopy are critical evidence of autophagic activity. In our investigation, transmission electron microscopy revealed that autophagosomes in the cytoplasm were significantly increased after baicalin treatment (Fig. 4B). In summary, our outcomes indicated that baicalin induces autophagosome generation. To further investigate the interaction between baicalin-induced autophagy and apoptosis, we added 3-MA, an autophagy inhibitor, to suppress autophagic activity. A cell viability assay revealed that pretreatment with 3-MA restored baicalin-medicated inhibition of cell viability (Fig. 4C). This was further substantiated by results showing that pretreatment with 3-MA reduced the percentage of baicalin-induced apoptotic cells from 26.8 ± 1.7 to 20.1 ± 1.3% (Fig. 4D). In summary, these results indicate that inhibition of autophagy attenuates baicalin-induced apoptosis.

### 3.5 Baicalin induces ROS and Ca^2+^ generation and blocks the PI3K/Akt/mTOR, ERK1/2 and β-catenin signaling pathways in OS cells

**Fig. 5:**
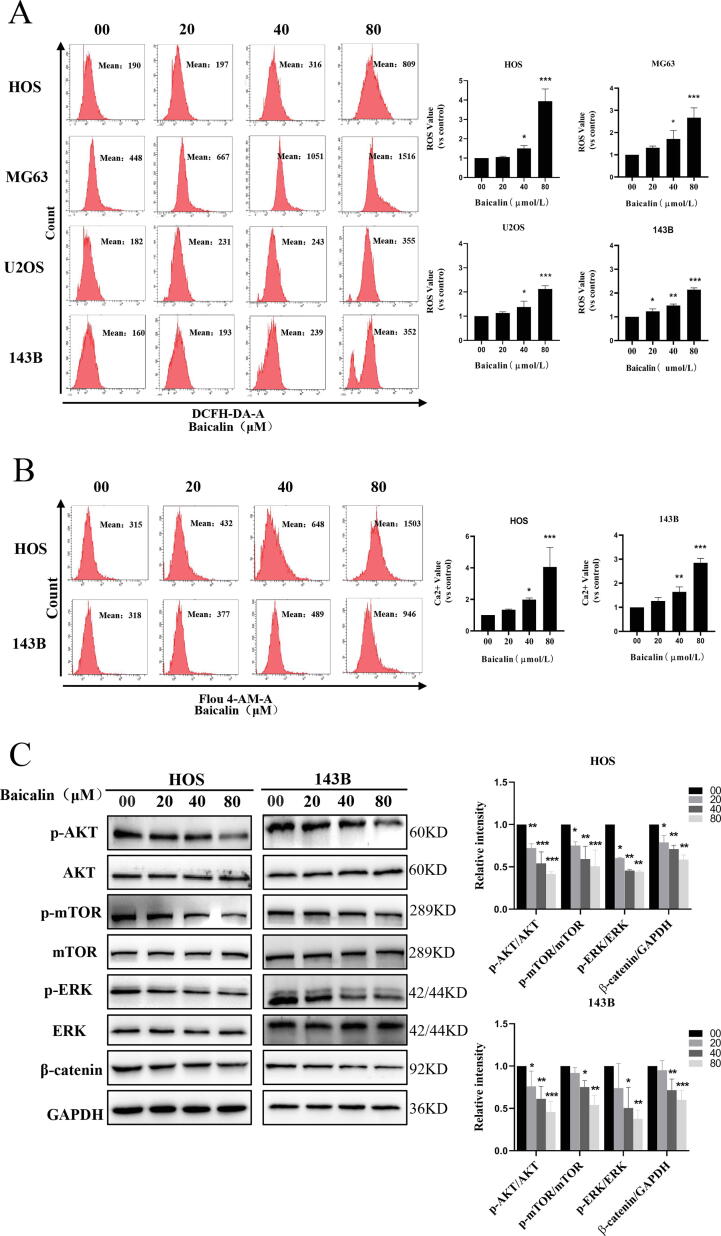
**Baicalin induces ROS and Ca^2+^ generation and inhibits the PI3K/Akt/mTOR, ERK1/2 and β-catenin pathways in OS cells.** Different concentrations of baicalin were used to treat OS cell for 48 h. **(A)** Flow cytometry analysis was used to assess ROS levels. **(B)** Intercellular Ca^2+^ was measured by determining Fluo-4 fluorescence using flow cytometry. **(C)** The signaling pathway protein levels of AKT, p-AKT, mTOR, p-mTOR, p-ERK, ERK, and β-catenin were measured by western blotting. *p < 0.05, **p < 0.01, ***p < 0.001 vs. control group.

ROS are intracellular and intercellular second messengers that are essential regulators of apoptosis and autophagy. ROS generation is primarily derived from the respiratory chain of mitochondria [43]. Our results showed that baicalin treatment sharply decreased the mitochondrial membrane potential in osteosarcoma cells. Therefore, we further speculated that the release of ROS in mitochondria would be induced by baicalin. To demonstrate this, we performed a ROS assay, which revealed that OS cells exposed to baicalin exhibited augmented intracellular ROS levels in a concentration-dependent manner. The levels of intracellular ROS increased by 3.9 ± 0.6-fold, 2.7 ± 0.5-fold, 2.1 ± 0.1-fold, and 2.2 ± 0.1-fold in the 80 µmol/L baicalin-treated group in HOS, MG63, U2OS and 143B cells, respectively, compared to controls (Fig. 5A). ROS and calcium ions are closely linked to each other through multiple mechanisms, and both inhibit the occurrence and development of tumors by mutual regulation. We then employed Fluo-4 to determine the intracellular Ca^2+^ concentration by flow cytometry. The results showed that intercellular Ca^2+^ concentration increased 4.1 ± 1.2-fold and 2.8 ± 0.2-fold after 48 h of baicalin treatment in HOS and 143B cells, respectively (Fig. 5B). To further investigate the anti-osteosarcoma mechanism of baicalin, we investigated its effects on the PI3K/Akt/mTOR, ERK1/2 and β-catenin signaling pathways. These signaling pathways regulate proliferation, invasion, apoptosis and autophagy. Our results revealed that baicalin decreased expression levels of β-catenin (Fig. 5C). Then, the results of our experiments demonstrated that baicalin inhibited AKT, mTOR and ERK1/2 protein phosphorylation (Fig. 5C). In summary, these findings reveal that baicalin activates ROS and Ca^2+^ and blocks the PI3K/Akt/mTOR, ERK1/2 and β-catenin pathways in osteosarcoma cells.

### 3.6 Baicalin induces apoptosis and autophagy in osteosarcoma cells by accumulating ROS to inhibit the PI3K/Akt/mTOR, ERK1/2 and β-catenin signaling pathways

**Fig. 6:**
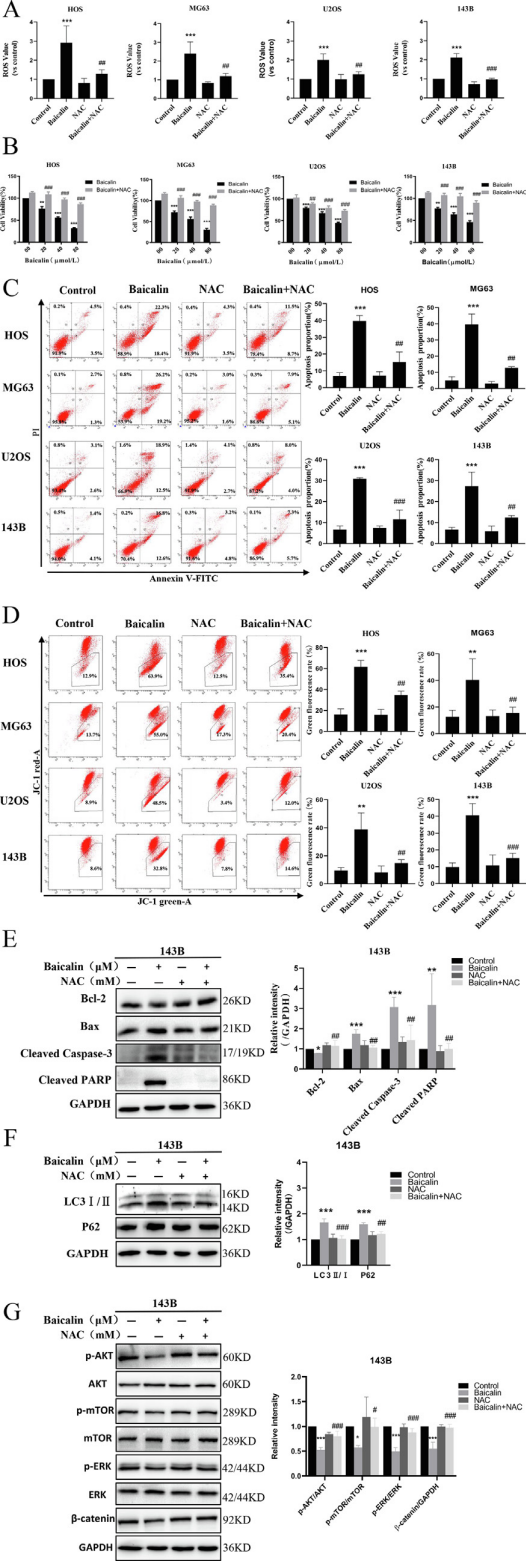
**Baicalin induces apoptosis and autophagy by accumulating ROS to suppress the PI3K/Akt/mTOR, ERK1/2 and β-catenin pathways in OS cells.** Osteosarcoma cells were pretreated with NAC for 1 h and then treated with baicalin for 48 h. **(A)** ROS content was assessed by flow cytometry analysis. **(B)** A CCK-8 assay was conducted to evaluate cell viability. **(C)** The percentage of apoptotic cells was evaluated by flow cytometry. **(D)** MMP was measured using flow cytometry. **(E, F)** Apoptosis-related protein and autophagy-related protein levels were measured by western blot analysis. **(G)** The signaling pathway protein levels of p-mTOR, mTOR, p-AKT, AKT, p-ERK, ERK, and β-catenin in 143B OS cells were measured by western blotting. *p < 0.05, **p < 0.01, ***p < 0.001 vs. control group. **^#^***p* < 0.05, **^##^***p* < 0.01, **^###^***p* < 0.001 vs. the baicalin-treated group.

To explore whether baicalin induces apoptosis and autophagy by accumulating ROS, we pretreated cells with 5 mM NAC, a ROS scavenger, to eliminate accumulating ROS induced by baicalin. The results showed that NAC pretreatment reduced baicalin-induced intracellular ROS generation (Fig. 6A). Remarkably, these results demonstrated that NAC pretreatment restored the inhibitory cell viability of baicalin (Fig. 6B). In addition, we demonstrated that baicalin-induced apoptosis was significantly decreased after NAC pretreatment (Fig. 6C). NAC pretreatment also reversed the decrease in mitochondrial potential induced by baicalin (Fig. 6D). These results reveal that NAC pretreatment reverses baicalin-induced expression of apoptosis-associated proteins (Fig. 6E), consistent with the above results. In terms of autophagy, the results showed that autophagy-related protein expression levels of LC3-Ⅱ and p62 were decreased after NAC pretreatment (Fig. 6F). Moreover, these results indicated that pretreatment with NAC reversed the baicalin-induced p-Akt, p-mTOR, p-ERK and β-catenin expression levels (Fig. 6G). Overall, these findings demonstrate that ROS negatively regulate the PI3K/Akt/mTOR, ERK1/2 and β-catenin signaling pathways.

### 3.7 Baicalin-induced ROS and Ca^2+^ interactions induce apoptosis in osteosarcoma

**Fig. 7:**
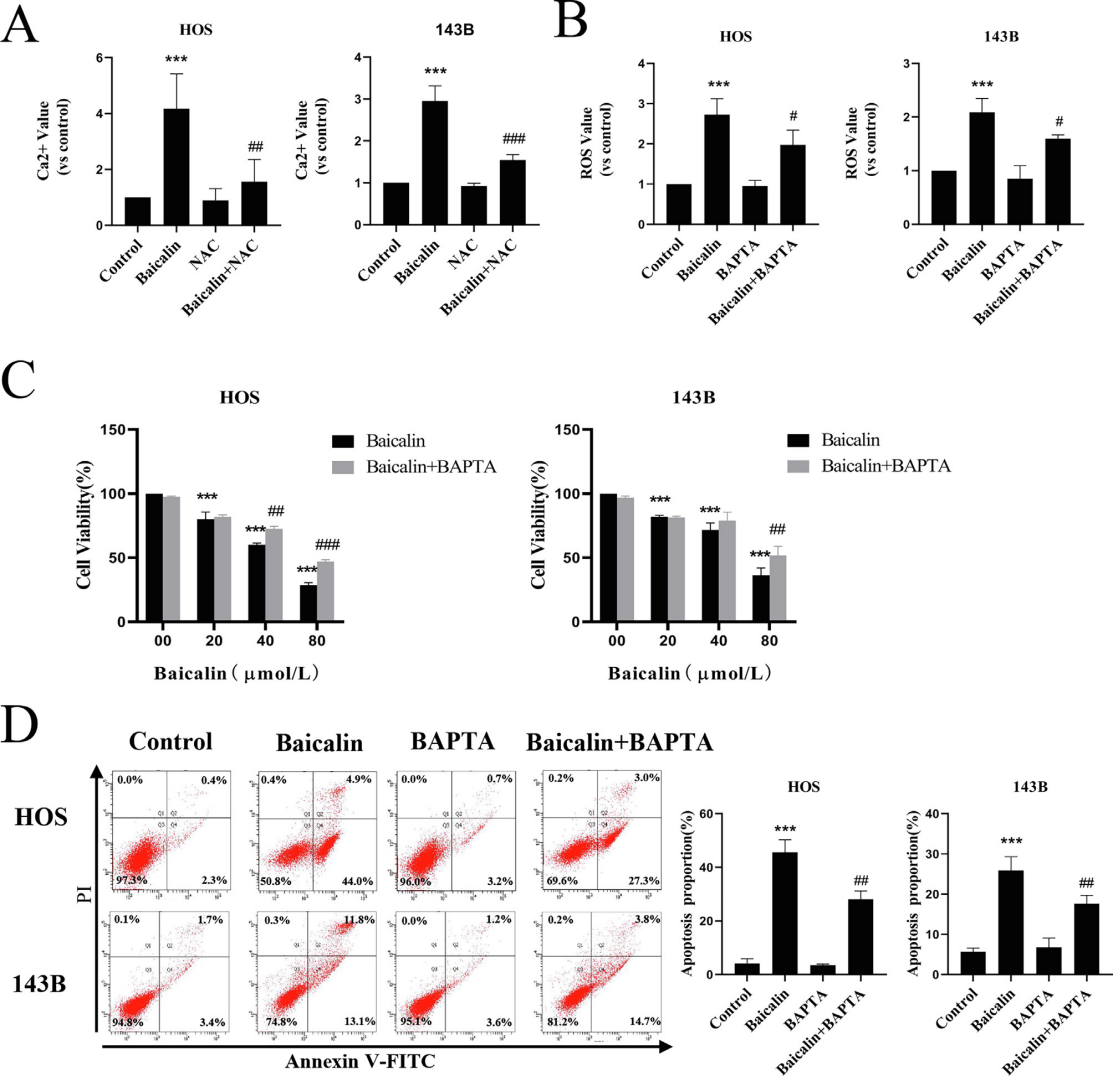
**Baicalin-induced ROS and Ca2^+^ interactions induce apoptosis**. Osteosarcoma cells were pretreated with BAPTA-AM or NAC for 1 h followed by treatment with baicalin for 48 h. **(A)** Fluo-4 AM fluorescence was used to assess the intercellular Ca^2+^ concentration using flow cytometry. **(B)** Flow cytometry was used to measure the content of ROS. **(C)** The CCK-8 assay was conducted to evaluate cell viability. **(D)** The apoptotic rate was evaluated by flow cytometry. *p < 0.05, **p < 0.01, ***p < 0.001 vs. control group. **^#^***p* < 0.05, **^##^***p* < 0.01, **^###^***p* < 0.001 vs. the baicalin-treated group.

Intercellular Ca^2+^ and ROS are stimulators of autophagy and apoptosis. However, whether Ca^2+^ is a stimulator of baicalin-induced apoptosis remains to be revealed. BAPTA-AM is designed to reduce Ca^2+^ overload due to its ability to penetrate the cell membrane and chelate free Ca^2+^ inside the cell. Our results revealed that pretreatment with BAPTA-AM (5 μM) decreased baicalin-induced ROS expression from 2.73 ± 0.4-fold to 2.09 ± 0.4-fold and from 2.1 ± 0.3-fold to 1.6 ± 0.1-fold in HOS and 143B cells, respectively (Fig. 7A). At the same time, pretreatment with NAC decreased intracellular Ca^2+^ concentrations from 4.12 ± 1.3-fold to 1.6 ± 0.8-fold and from 3.0 ± 0.4-fold to 1.5 ± 0.1-fold (Fig. 7B). Moreover, our observations revealed that BAPTA pretreatment significantly restored cell viability (Fig. 7C) and decreased the apoptotic ratio from 45.6 ± 4.7 to 28.2 ± 3.0% and from 25.9 ± 3.4% to 17.6 ± 2.0% in HOS and 143B cells, respectively, compared to baicalin alone (Fig. 7D). In summary, these findings reveal that baicalin-induced ROS and Ca^2+^ interactions cause apoptosis in OS cells.

### 3.8 The specific interactions between PI3Kγ and baicalin

**Fig. 8:**
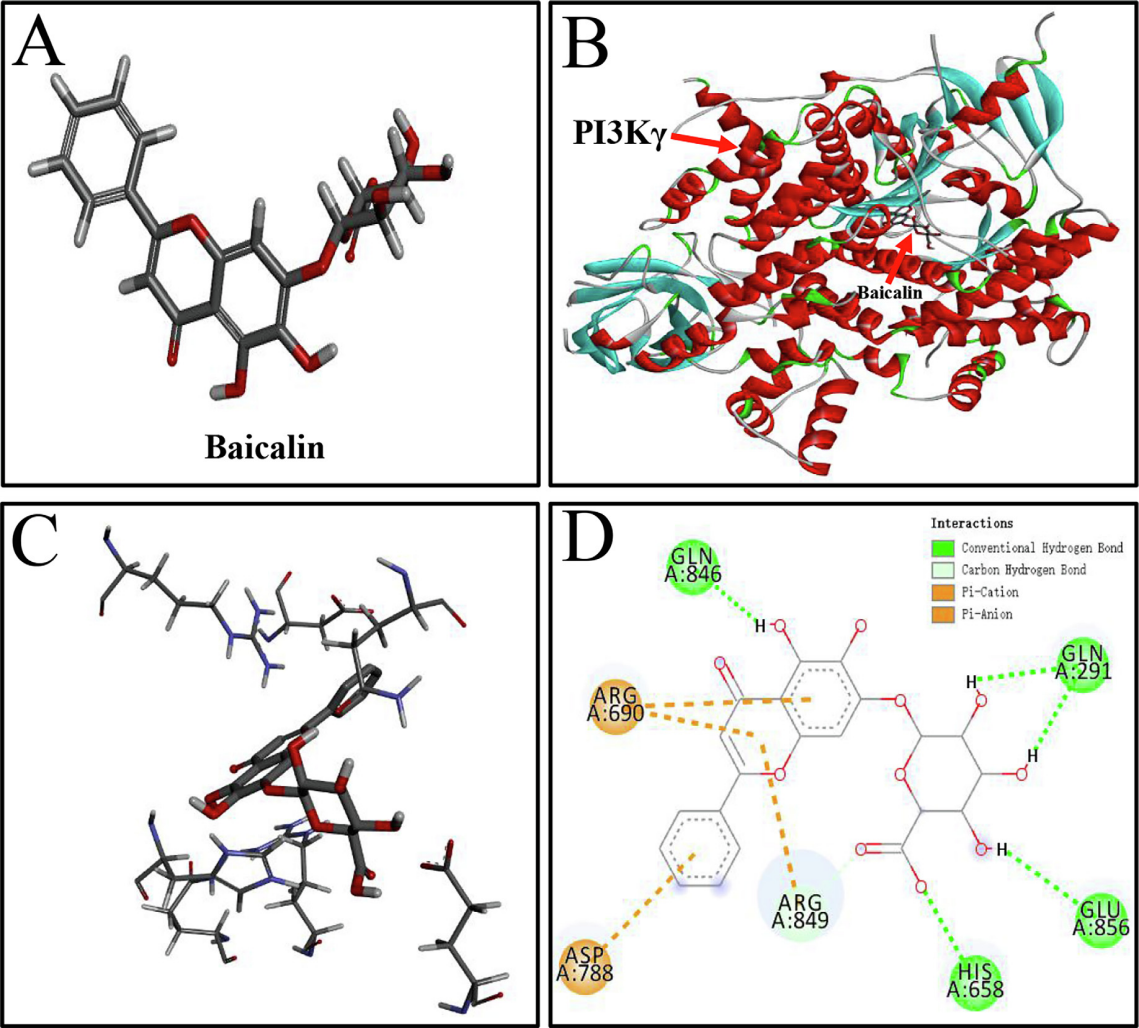
**The intermolecular interactions between PI3Kγ and baicalin. (A)** The 3D structure of baicalin. **(B)** Interaction sites between PI3Kγ and baicalin in the 3D diagram**. (C)** Specific interactions between baicalin and PI3K γ-acid residues in the 3D structural diagram. **(D)** Hydrogen-bonding interactions between PI3Kγ and baicalin in the 2D diagram. Conventional hydrogen bonds are represented by green dashes. Pi-Cation and Pi-Anion are represented by brown dashes. (For interpretation of the references to color in this figure legend, the reader is referred to the web version of this article.)

To explore the specific interactions between baicalin and PI3Kγ, molecular docking was applied to simulate their binding. The results demonstrated that the binding energy of PI3Kγ and baicalin was −9.7 kcal/mol. The binding energy of PI3Kγ and a PI3Kγ inhibitor (PDB ID: 4XZ4) was −7.5 kcal/mol. After importing software to simulate molecular docking, the three-dimensional structure of baicalin (Fig. 8A) was processed by hydrogenation, charge calculation, and charge assignment. Molecular docking of PI3Kγ and baicalin revealed that baicalin was encapsulated by the internal central cavity of PI3Kγ and docked at the PI3Kγ active site (Fig. 8B). The specific interactions between baicalin and amino acid residues of PI3Kγ in the 3D schematic diagram (Fig. 8C) and 2D diagram of interactions (Fig. 8D) revealed that PI3Kγ was linked to baicalin through four hydrogen bonds, indicating that baicalin binds human-derived PI3Kγ with high affinity. Altogether, these results suggest that the specific interaction between PI3Kγ and baicalin exerts an anti-osteosarcoma effect.

## 4 Discussion

Osteosarcoma is the most common form of bone malignancy and derives from primitive bone-forming mesenchymal cells. At present, surgical resection is the primary means of treating patients with osteosarcoma [44]. Remarkably, the active ingredients of natural compounds also have an essential function in osteosarcoma treatment [45]. The present work demonstrates that baicalin suppresses the proliferative capacity and invasive ability of human osteosarcoma cells. Cell cycle arrest can inhibit tumor cell proliferation. According to the results, baicalin increased the S-phase ratio in osteosarcoma cells. Furthermore, research revealed that baicalin inhibits c-Myc, cyclin A2, Cdk2 and cyclin E1 expression, which is crucial for proliferation and the cell cycle. These results suggest that baicalin induces S-phase accumulation and inhibits proliferation in osteosarcoma cells.

Cell cycle arrest can trigger cell death [16], [46]. Apoptosis is triggered by multiple signaling pathways [47], [48]. Most conventional chemotherapeutic agents depend on the activation of apoptotic pathways to exert their anticancer effects. Recent evidence indicates that endogenous and exogenous pathways are engaged in regulating apoptosis [49], [50]. Moreover, mitochondria play a significant role in the endogenous pathway [51]. PARP is a marker of apoptosis that repairs damaged nuclear DNA and is inactivated by cleavage of caspase-3 [52]. Our results revealed that treatment with baicalin reduces mitochondrial membrane potential (MMP). By observing increased Bax, cleaved caspase-3 and cleaved PARP expression in osteosarcoma cells, we demonstrated that baicalin induced apoptosis. These results showed that baicalin treatment decreased the expression of Bcl-2, which also supported the induction of apoptosis. Since the activation of Bax protein can lead to a decrease in mitochondrial membrane potential [53], our findings indicated that baicalin triggers a cascade of apoptotic mitochondrial pathways. These outcomes demonstrated that baicalin disrupts the mitochondrial membrane integrity of osteosarcoma cells and activates apoptosis.

In addition to apoptosis, autophagy plays an essential role in regulating cell death. Throughout the autophagic process, cellular materials and organelles form autophagosomes, which are eventually digested in lysosomes [54], [55]. When autophagy is triggered, LC3 transforms from its soluble LC3-I form to the lipolytic LC3-II form and binds to autophagic vesicles to form autophagosomes [56]. Our investigation revealed an increase in the LC3-II/LC3-I ratio in baicalin-treated cells, indicating that autophagy was triggered and autophagosome formation occurs. The dynamic process of autophagy includes the formation of autophagosomes, fusion of autophagosomes with lysosomes and lysosomal degradation. During lysosomal degradation, substrate-bound p62 is degraded by proteolytic enzymes. At the same time, blockade of the downstream process of autophagy leads to accumulation of p62. Therefore, elevated p62 expression is generally considered a marker of downstream inhibition of autophagy [57], [58]. These results indicated that baicalin treatment increased the expression of p62. Taken together, these results indicate an increase in the LC3-II/LC3-I ratio and p62 expression in baicalin-treated cells, indicating that baicalin treatment induces autophagosome formation but blocks dynamic processes downstream of autophagy. Another reason for the accumulation of p62 may be correlated to the nature of the protein itself: p62 is an oxidative stress protein whose expression levels are markedly upregulated under stress conditions, and this process is primarily regulated by the transcription factor EB [59]. Accumulating evidence indicates that autophagy can both protect and damage cells in different cancer settings [60]. The above study indicates that the baicalin-induced autophagic process induces the formation of autophagosomes rather than the completion of autophagy. However, this incomplete process recruits caspase-8 to initiate the caspase-3 cascade reaction, causing cell death [61]. Furthermore, these results revealed that baicalin-induced apoptosis was attenuated by 3-MA. We presumed that the primary reason for this is that baicalin-induced autophagosomes further triggered caspase-3-dependent apoptosis. Therefore, 3-MA suppressed the initial phase of autophagy, leading to a decrease in apoptosis and confirming the above speculation.

ROS are byproducts of mitochondrial metabolism. Cancer cells contain high basal ROS levels, which cause sensitizes cells to excessive oxidative stress. Therefore, excessive ROS are also deadly to osteosarcoma cells [62]. Research has indicated that multiple chemotherapeutic agents induce apoptosis and autophagy in tumor cells through ROS generation [30]. Interestingly, baicalin has antioxidant properties in human umbilical vein endothelial cells, fibroblasts, and glial cells [63], [64], [65]. However, it promotes ROS generation in a variety of tumor cells, inducing tumor cell apoptosis [66], [67]. At the same time, such a phenomenon is not uncommon in anticancer drugs and may be attributed to different mechanisms or targets for increasing ROS in different types of cells. Our previous data showed that baicalin dramatically increased ROS production in osteosarcoma cells. In this study, baicalin-induced apoptosis and autophagy were significantly reversed after ROS removal by NAC. Therefore, these results reveal that baicalin induces high ROS production to trigger apoptosis and autophagy. Increased intracellular Ca^2+^ content aggravates endoplasmic reticulum stress and disturbs mitochondrial membrane potential, leading to mitochondria-dependent apoptosis [68]. Several investigations have suggested that cytoplasmic Ca^2+^ significantly induces both autophagy and apoptosis [69]. Because ROS and Ca^2+^ are tightly linked in many pathways, it is not surprising that increasing mitochondrial Ca^2+^ uptake leads to enhanced mitochondrial ROS production in cancer cells [39]. In our work, we discovered that baicalin significantly increased intracellular Ca^2+^ concentrations in osteosarcoma cells. Baicalin-induced apoptosis and ROS production were attenuated when cells were pretreated with BAPTA-AM. Notably, NAC pretreatment decreased the Ca^2+^ concentration. However, our work did not focus on intercellular Ca^2+^ in autophagy or the associated signaling pathways. An abundance of data indicates that the PI3K/Akt/mTOR, ERK1/2 and β-catenin signaling pathways are closely related to proliferation, apoptosis and autophagy [70], [71], [72]. In addition, extensive studies have reported that accumulating ROS inhibits the PI3K/Akt/mTOR, ERK1/2 and β-catenin pathways, leading to autophagy and apoptosis [36], [37], [73]. This research revealed a remarkable reduction in p-Akt, p-mTOR, p-ERK1/2, and β-catenin in response to baicalin treatment. Furthermore, NAC pretreatment restored these pathways inhibited by baicalin. In summary, these results suggest that baicalin induces apoptosis and autophagy in OS cells by inducing ROS to inhibit the PI3K/Akt/mTOR, ERK1/2 and β-catenin signaling pathways.

All of these results suggest that baicalin suppresses osteosarcoma cells. However, the relationship between baicalin’s chemical components and targets remains unclear. PI3Kγ is a class of PI3Ks [74]. Data have indicated that blocking PI3Kγ improves tumor sensitivity to chemotherapeutic agents and enhances cancer immunotherapy to eradicate tumors [75]. Therefore, we speculated that baicalin may target PI3Kγ. To investigate the specific interaction between PI3Kγ and baicalin, we performed molecular docking simulations. The results showed that baicalin strongly interacts with the inner lumen of PI3Kγ, suggesting tight binding of PI3Kγ to baicalin. The underlying direct binding suggested that the combination of PI3Kγ and baicalin may inhibit the downstream effectors AKT and mTOR. However, further experimental evidence is needed to verify our results. In summary, baicalin exerts an anti-osteosarcoma effect by targeting PI3Kγ. This discovery provides a new candidate target drug for osteosarcoma treatment and proposes a new perspective on the mechanism underlying the anti-osteosarcoma effect of baicalin.

## 5 Conclusion

**Fig. 9:**
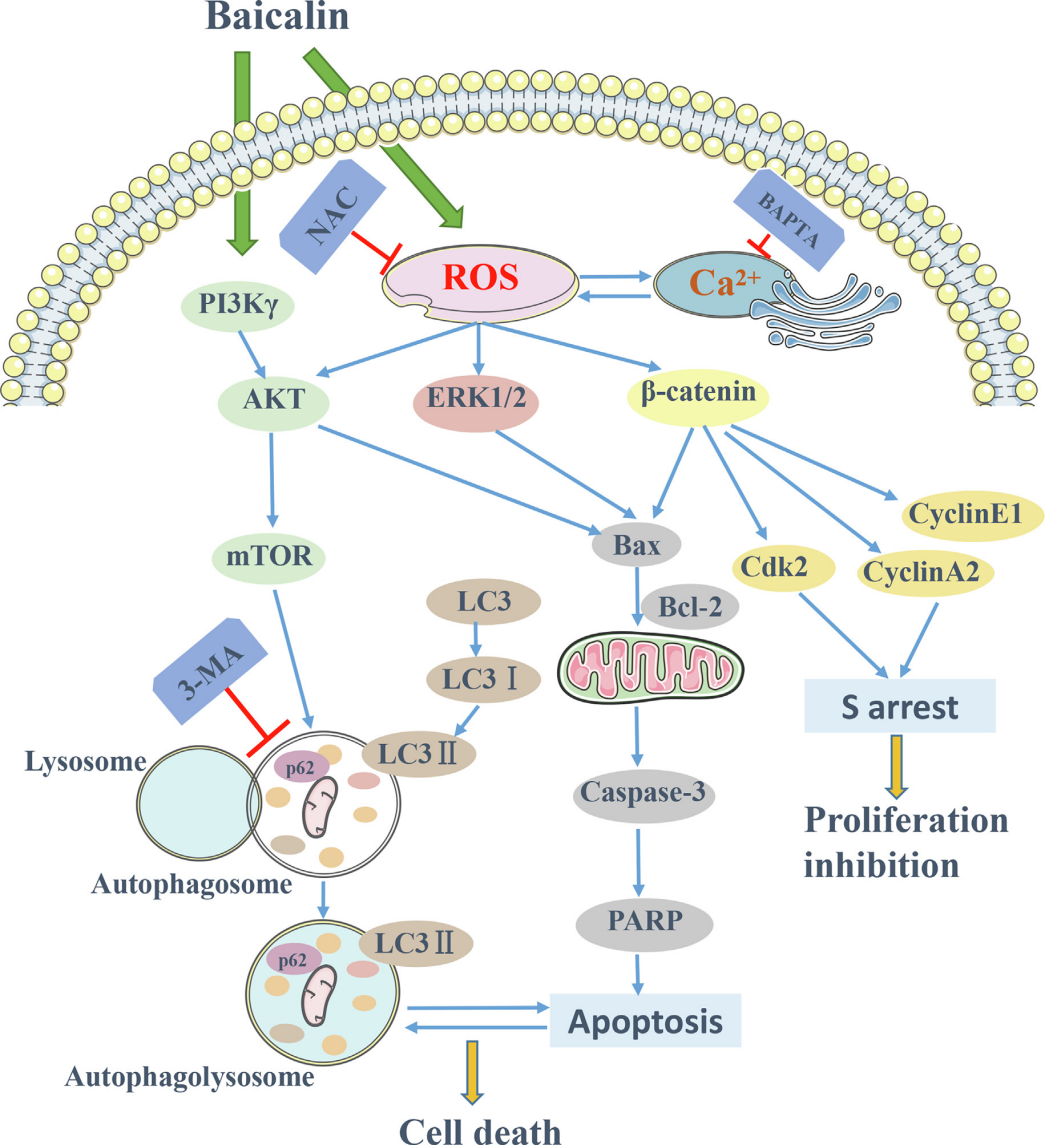
Mechanism of baicalin-induced apoptosis and autophagy in osteosarcoma cells.

Our results demonstrated that apoptosis and autophagy are induced by baicalin in osteosarcoma cells. Next, we examined the relationship between baicalin-induced apoptosis and autophagy. Moreover, we further proposed that the anti-osteosarcoma effect of baicalin is mediated by accumulating ROS to inhibit the PI3K/Akt/mTOR, ERK1/2 and β-catenin pathways. Baicalin also exerts an anti-osteosarcoma effect by targeting PI3Kγ. Due to its excellent anticancer efficacy and relevant mechanism (Fig. 9), we recommend considering baicalin as a possible new anticancer agent against osteosarcoma.

## Author contributions

BW, HP, and LS proposed the idea. HP, ZP, and QT explore the experiments. HP, TW, ZZ, XP, and ZP analyzed the results. BW, HP, LS, and TW finished the paper.

## CRediT authorship contribution statement

**He Pang:** Conceptualization, Formal analysis, Investigation, Methodology, Software, Writing – original draft. **Tingrui Wu:** Formal analysis, Methodology, Writing – original draft, Writing – review & editing. **Zhonghua Peng:** Investigation, Resources, Visualization. **Qichao Tan:** Investigation, Validation. **Xin Peng:** Software, Validation. **Zeyu Zhan:** Formal analysis, Visualization. **Lijun Song:** Conceptualization, Methodology, Resources, Writing – review & editing. **Bo Wei:** Conceptualization, Funding acquisition, Resources, Supervision, Writing – review & editing.

## Declaration of Competing Interest

The authors declare that they have no known competing financial interests or personal relationships that could have appeared to influence the work reported in this paper.

## References

[1] Lilienthal I., Herold N. (2020). Targeting molecular mechanisms underlying treatment efficacy and resistance in osteosarcoma: a review of current and future strategies. Int. J. Mol. Sci..

[2] Raymond A., Jaffe N. (2009). Osteosarcoma multidisciplinary approach to the management from the pathologist's perspective. Cancer Treat. Res..

[3] Gelberg K., Fitzgerald E., Hwang S., Dubrow R. (1997). Growth and development and other risk factors for osteosarcoma in children and young adults. Int. J. Epidemiol..

[4] Mirabello L., Pfeiffer R., Murphy G., Daw N., Patiño-Garcia A., Troisi R., Hoover R., Douglass C., Schüz J., Craft A., Savage S. (2011). Height at diagnosis and birth-weight as risk factors for osteosarcoma. Cancer Causes & Control.

[5] Berner K., Johannesen T.B., Berner A., Haugland H.K., Bjerkehagen B., Bøhler P.J., Bruland S.Ø. (2015). Time-trends on incidence and survival in a nationwide and unselected cohort of patients with skeletal osteosarcoma. Acta Oncol..

[6] Grinberg S., Posta A., Weber K., Wilson R. (2020). Limb salvage and reconstruction options in osteosarcoma. Adv. Exp. Med. Biol..

[7] L. Pan, K. Cho, I. Yi, C. To, D. Chen, C. Do, Baicalein, Baicalin, and Wogonin: Protective Effects against Ischemia-Induced Neurodegeneration in the Brain and Retina, Oxidative medicine and cellular longevity 2021 (2021) 8377362. 10.1155/2021/8377362 PMC8263226 34306315

[8] Ma L., Wu F., Shao Q., Chen G., Xu L., Lu F. (2021). Baicalin Alleviates Oxidative Stress and Inflammation in Diabetic Nephropathy via Nrf2 and MAPK Signaling Pathway. Drug Des. Dev. Therapy.

[9] Wang B., Huang T., Fang Q., Zhang X., Yuan J., Li M., Ge H. (2020). Bone-protective and anti-tumor effect of baicalin in osteotropic breast cancer via induction of apoptosis. Breast Cancer Res. Treat..

[10] Gong W.-y., Zhao Z.-X., Liu B.-J., Lu L.-W., Dong J.-C. (2017). Exploring the chemopreventive properties and perspectives of baicalin and its aglycone baicalein in solid tumors. Eur. J. Med. Chem..

[11] Zhu Y., Fang J., Wang H., Fei M., Tang T., Liu K., Niu W., Zhou Y. (2018). Baicalin suppresses proliferation, migration, and invasion in human glioblastoma cells via Ca-dependent pathway. Drug Des. Dev. Therapy.

[12] Wang Y., Wang H., Zhou R., Zhong W., Lu S., Ma Z., Chai Y. (2017). Baicalin inhibits human osteosarcoma cells invasion, metastasis, and anoikis resistance by suppressing the transforming growth factor-β1-induced epithelial-to-mesenchymal transition. Anticancer Drugs.

[13] Wan D., Ouyang H. (2018). Baicalin induces apoptosis in human osteosarcoma cell through ROS-mediated mitochondrial pathway. Nat. Prod. Res..

[14] Liu Y., Hong Z., Chen P., Wang J., Zhou Y., Huang J. (2019). Baicalin inhibits growth and induces apoptosis of human osteosarcoma cells by suppressing the AKT pathway. Oncol. Lett..

[15] Lan H., Wang H., Gao M., Luo G., Zhang J., Yi E., Liang C., Xiong X., Chen X., Wu Q., Chen R., Lin B., Qian D., Hong W. (2021). Analysis and construction of a competitive endogenous RNA regulatory network of baicalin-induced apoptosis in human osteosarcoma cells. Biomed Res. Int..

[16] T. Osawa, D. Davies, J. Hartley, Mechanism of cell death resulting from DNA interstrand cross-linking in mammalian cells, Cell Death & Disease 2 (2011) e187. 10.1038/cddis.2011.70 PMC3181417 21814285

[17] N. Susanti, D. Tjahjono, Cyclin-dependent kinase 4 and 6 inhibitors in cell cycle dysregulation for breast cancer treatment, Molecules (Basel, Switzerland) 26(15) (2021). 10.3390/molecules26154462 PMC8348313 34361615

[18] Xie T., Ren H.Y., Lin H.Q., Mao J.P., Zhu T., Wang S.D., Ye Z.M. (2016). Sinomenine prevents metastasis of human osteosarcoma cells via S phase arrest and suppression of tumor-related neovascularization and osteolysis through the CXCR4-STAT3 pathway. Int. J. Oncol..

[19] Yin Z., Chen E., Cai X., Gong E., Li Y., Xu C., Ye Z., Cao Z., Pan J. (2021). Baicalin attenuates XRCC1-mediated DNA repair to enhance the sensitivity of lung cancer cells to cisplatin. J. Receptor Signal Transduction Res..

[20] Booth L.A., Roberts J.L., Dent P. (2020). The role of cell signaling in the crosstalk between autophagy and apoptosis in the regulation of tumor cell survival in response to sorafenib and neratinib. Semin. Cancer Biol..

[21] Suzanne M., Steller H. (2013). Shaping organisms with apoptosis. Cell Death Differ..

[22] Wu M., Fu T., Chen J., Lin Y., Yang J., Zhuang S. (2020). LncRNA GOLGA2P10 is induced by PERK/ATF4/CHOP signaling and protects tumor cells from ER stress-induced apoptosis by regulating Bcl-2 family members. Cell Death Dis..

[23] White E., Lattime E.C., Guo J.Y. (2021). Autophagy regulates stress responses, metabolism, and anticancer immunity. Trends Cancer.

[24] Poillet-Perez L., Sarry J.-E., Joffre C. (2021). Autophagy is a major metabolic regulator involved in cancer therapy resistance. Cell Rep..

[25] Zhang Z., Shi J., Nice E., Huang C., Shi Z. (2021). The multifaceted role of flavonoids in cancer therapy: leveraging autophagy with a double-edged sword. Antioxidants (Basel Switzerland).

[26] Yuan Y., Jiang N., Li Z., Song Z., Yang Z., Xue W., Zhang X., Du Y. (2019). Polyphyllin VI induces apoptosis and autophagy in human osteosarcoma cells by modulation of ROS/JNK activation. Drug Des. Dev. Therapy.

[27] Decuypere J.-P., Welkenhuyzen K., Luyten T., Ponsaerts R., Dewaele M., Molgó J., Agostinis P., Missiaen L., De Smedt H., Parys J.B., Bultynck G. (2011). Ins(1,4,5)P3 receptor-mediated Ca2+ signaling and autophagy induction are interrelated. Autophagy.

[28] Li B., Zhou P., Xu K., Chen T., Jiao J., Wei H., Yang X., Xu W., Wan W., Xiao J. (2020). Baicalin induces cell cycle arrest, apoptosis and autophagy through ROS/JNK signaling pathway in human osteosarcoma. Int. J. Biol. Sci..

[29] Law B., Michelangeli F., Qu Y., Xu S., Han Y., Mok S., Dias I., Javed M., Chan W., Xue W., Yao X., Zeng W., Zhang H., Wang J., Liu L., Wong V. (2019). Neferine induces autophagy-dependent cell death in apoptosis-resistant cancers via ryanodine receptor and Ca-dependent mechanism. Sci. Rep..

[30] Tang J.-Y., Ou-Yang F.u., Hou M.-F., Huang H.-W., Wang H.-R., Li K.-T., Fayyaz S., Shu C.-W., Chang H.-W. (2019). Oxidative stress-modulating drugs have preferential anticancer effects - involving the regulation of apoptosis, DNA damage, endoplasmic reticulum stress, autophagy, metabolism, and migration. Semin. Cancer Biol..

[31] Zahra K., Lefter R., Ali A., Abdellah E., Trus C., Ciobica A., Timofte D. (2021). The involvement of the oxidative stress status in cancer pathology: a double view on the role of the antioxidants. Oxid. Med. Cell. Longevity.

[32] Trachootham D., Alexandre J., Huang P. (2009). Targeting cancer cells by ROS-mediated mechanisms: a radical therapeutic approach?. Nat. Rev. Drug Discovery.

[33] Hayes J.D., Dinkova-Kostova A.T., Tew K.D. (2020). Oxidative stress in cancer. Cancer Cell.

[34] Huynh D.T.N., Jin Y., Myung C.-S., Heo K.-S. (2021). Ginsenoside Rh1 induces MCF-7 cell apoptosis and autophagic cell death through ROS-mediated Akt signaling. Cancers.

[35] Thévenod F., Lee W. (2013). Cadmium and cellular signaling cascades: interactions between cell death and survival pathways. Arch. Toxicol..

[36] Hu K., Li W., Sun M., Zhang S., Fan C., Wu Q., Zhu W., Xu X. (2015). Cadmium induced apoptosis in MG63 cells by increasing ROS, activation of p38 MAPK and inhibition of ERK 1/2 pathways. Cell. Physiol. Biochem..

[37] Zhang C., Huang C., Yang P., Li C., Li M. (2021). Eldecalcitol induces apoptosis and autophagy in human osteosarcoma MG-63 cells by accumulating ROS to suppress the PI3K/Akt/mTOR signaling pathway. Cell. Signal..

[38] Siqueira E.d.S., Concato V.M., Tomiotto-Pellissier F., Silva T.F., Bortoleti B.T.d.S., Gonçalves M.D., Costa I.N., Junior W.A.V., Pavanelli W.R., Panis C., Mantovani M.S., Miranda-Sapla M.M., Conchon-Costa I. (2021). Trans-chalcone induces death by autophagy mediated by p53 up-regulation and β-catenin down-regulation on human hepatocellular carcinoma HuH7.5 cell line. Phytomed.: Int. J. Phytotherapy Phytopharmacol..

[39] Madreiter-Sokolowski C., Thomas C., Ristow M. (2020). Interrelation between ROS and Ca in aging and age-related diseases. Redox Biol..

[40] Kuchay S., Giorgi C., Simoneschi D., Pagan J., Missiroli S., Saraf A., Florens L., Washburn M., Collazo-Lorduy A., Castillo-Martin M., Cordon-Cardo C., Sebti S., Pinton P., Pagano M. (2017). PTEN counteracts FBXL2 to promote IP3R3- and Ca-mediated apoptosis limiting tumour growth. Nature.

[41] Xu C., Chen S., Xu M., Chen X., Wang X., Zhang H., Dong X., Zhang R., Chen X., Gao W., Huang S., Chen L. (2021). Cadmium impairs autophagy leading to apoptosis by Ca-dependent activation of JNK signaling pathway in neuronal cells. Neurochem. Res..

[42] Yin Q.i., Chen H., Ma R.-H., Zhang Y.-Y., Liu M.-M., Thakur K., Zhang J.-G., Wei Z.-J. (2021). Ginsenoside CK induces apoptosis of human cervical cancer HeLa cells by regulating autophagy and endoplasmic reticulum stress. Food Funct..

[43] Li D., Ding Z., Du K., Ye X., Cheng S. (2021). Reactive oxygen species as a link between antioxidant pathways and autophagy. Oxid. Med. Cell. Longevity.

[44] Cascini C., Chiodoni C. (2021). The immune landscape of osteosarcoma: implications for prognosis and treatment response. Cells.

[45] Xiang Y., Guo Z., Zhu P., Chen J., Huang Y. (2019). Traditional Chinese medicine as a cancer treatment: modern perspectives of ancient but advanced science. Cancer Med..

[46] Schwartz G., Shah M. (2005). Targeting the cell cycle: a new approach to cancer therapy. J. Clin. Oncol..

[47] Khan I., Yousif A., Chesnokov M., Hong L., Chefetz I. (2021). A decade of cell death studies: breathing new life into necroptosis. Pharmacol. Ther..

[48] Strasser A., Vaux D.L. (2020). Cell death in the origin and treatment of cancer. Mol. Cell.

[49] McArthur K., Kile B. (2018). Apoptotic caspases: multiple or mistaken identities?. Trends Cell Biol..

[50] Zhang J.-H., Zhang Y., Herman B. (2003). Caspases, apoptosis and aging. Ageing Res. Rev..

[51] Burke P.J. (2017). Mitochondria, bioenergetics and apoptosis in cancer. Trends Cancer.

[52] Nury T., Zarrouk A., Yammine A., Mackrill J., Vejux A., Lizard G. (2021). Oxiapoptophagy: a type of cell death induced by some oxysterols. Br. J. Pharmacol..

[53] Luna-Vargas M., Chipuk J. (2016). Physiological and pharmacological control of BAK, BAX, and beyond. Trends Cell Biol..

[54] Klionsky D.J., Emr S.D. (2000). Autophagy as a regulated pathway of cellular degradation. Science.

[55] Nakatogawa H. (2020). Mechanisms governing autophagosome biogenesis. Nat. Rev. Mol. Cell Biol..

[56] Onorati A.V., Dyczynski M., Ojha R., Amaravadi R.K. (2018). Targeting autophagy in cancer. Cancer.

[57] Yue Z., Guan X., Chao R., Huang C., Li D., Yang P., Liu S., Hasegawa T., Guo J., Li M. (2019). Diallyl disulfide induces apoptosis and autophagy in human osteosarcoma MG-63 Cells through the PI3K/Akt/mTOR pathway. Molecules (Basel Switzerland).

[58] Lamark T., Svenning S., Johansen T. (2017). Regulation of selective autophagy: the p62/SQSTM1 paradigm. Essays Biochem..

[59] Emanuele S., Lauricella M., D'Anneo A., Carlisi D., De Blasio A., Di Liberto D., Giuliano M. (2020). OncoJanusp62: friend or Foe? Evidences for and roles. Int. J. Mol. Sci..

[60] Miller D., Thorburn A. (2021). Autophagy and organelle homeostasis in cancer. Dev. Cell.

[61] Young M., Takahashi Y., Khan O., Park S., Hori T., Yun J., Sharma A., Amin S., Hu C., Zhang J., Kester M., Wang H. (2012). Autophagosomal membrane serves as platform for intracellular death-inducing signaling complex (iDISC)-mediated caspase-8 activation and apoptosis. J. Biol. Chem..

[62] Huang R., Chen H., Liang J., Li Y., Yang J., Luo C., Tang Y., Ding Y., Liu X., Yuan Q., Yu H., Ye Y., Xu W., Xie X. (2021). Dual role of reactive oxygen species and their application in cancer therapy. J. Cancer.

[63] Zhou B., Yin H., Xu Y., Wu D., Zhang Z., Yin Z., Permatasari F., Luo D. (2012). Baicalin protects human skin fibroblasts from ultraviolet A radiation-induced oxidative damage and apoptosis. Free Radical Res..

[64] Song X., Gong Z., Liu K., Kou J., Liu B., Liu K. (2020). Baicalin combats glutamate excitotoxicity via protecting glutamine synthetase from ROS-induced 20S proteasomal degradation. Redox Biol..

[65] Tsai C., Tsai C., Chang W., Lin J., Hsia T., Bau D. (2021). Protective effects of baicalin on arsenic trioxide-induced oxidative damage and apoptosis in human umbilical vein endothelial cells. In vivo (Athens, Greece).

[66] Zakki S., Cui Z., Sun L., Feng Q., Li M., Inadera H. (2018). Baicalin Augments Hyperthermia-Induced Apoptosis in U937 Cells and Modulates the MAPK Pathway via ROS Generation. Cell. Physiol. Biochem..

[67] Lan M., Kong Z., Liu F., Zou T., Li L., Cai T., Huaqin T., Cai Y. (2021). Activating caspase-8/Bid/ROS signaling to promote apoptosis of breast cancer cells by folate-modified albumin baicalin-loaded nanoparticles. Nanotechnology.

[68] Rimessi A., Bonora M., Marchi S., Patergnani S., Marobbio C., Lasorsa F., Pinton P. (2013). Perturbed mitochondrial Ca2+ signals as causes or consequences of mitophagy induction. Autophagy.

[69] Høyer-Hansen M., Bastholm L., Szyniarowski P., Campanella M., Szabadkai G., Farkas T., Bianchi K., Fehrenbacher N., Elling F., Rizzuto R., Mathiasen I., Jäättelä M. (2007). Control of macroautophagy by calcium, calmodulin-dependent kinase kinase-beta, and Bcl-2. Mol. Cell.

[70] He Y., Shi Y., Yang Y., Huang H., Feng Y., Wang Y., Zhan L., Wei B. (2021). Chrysin induces autophagy through the inactivation of the ROS-mediated Akt/mTOR signaling pathway in endometrial cancer. Int. J. Mol. Med..

[71] Tsai C., Ko H., Huang C., Lin C., Chiou S., Su Y., Lieu A., Loh J., Kwan A., Chuang T., Hong Y. (2021). Ionizing radiation induces resistant glioblastoma stem-like cells by promoting autophagy via the Wnt/β-Catenin Pathway. Life (Basel, Switzerland).

[72] Reddy D., Ghosh P., Kumavath R. (2019). Strophanthidin Attenuates MAPK, PI3K/AKT/mTOR, and Wnt/β-Catenin Signaling Pathways in Human Cancers. Front. Oncol..

[73] Zhu J., Li X., Liang C., Zhou X., Ge M., Chen Y., Jin J., Yin J., Xu H., Xie C., Zhong C. (2021). Apatinib suppresses lung cancer stem-like cells by complex interplay between β-catenin signaling and mitochondrial ROS accumulation. Cell Death Discovery.

[74] Ediriweera M., Tennekoon K., Samarakoon S. (2019). Role of the PI3K/AKT/mTOR signaling pathway in ovarian cancer: Biological and therapeutic significance. Semin. Cancer Biol..

[75] Kaneda M., Cappello P., Nguyen A., Ralainirina N., Hardamon C., Foubert P., Schmid M., Sun P., Mose E., Bouvet M., Lowy A., Valasek M., Sasik R., Novelli F., Hirsch E., Varner J. (2016). Macrophage PI3Kγ drives pancreatic ductal adenocarcinoma progression. Cancer Discovery.

